# ShenLian Extract Enhances TGF-β Functions in the Macrophage-SMC Unit and Stabilizes Atherosclerotic Plaques

**DOI:** 10.3389/fphar.2021.669730

**Published:** 2021-05-28

**Authors:** Li Liu, Qi Li, Jie Yin, Zheng Zhao, Lidong Sun, Qingsen Ran, Xinke Du, Yajie Wang, Yujie Li, Qing Yang, Ying Chen, Xiaogang Weng, Weiyan Cai, Xiaoxin Zhu

**Affiliations:** ^1^ Institute of Chinese Materia Medica, China Academy of Chinese Medical Sciences, Beijing, China; ^2^ Leiden University, Leiden, Netherlands

**Keywords:** atherosclerotic plaques stability, transforming growth factor-β, smooth muscle cells phenotype switching, macrophage polarization, shenlian extract

## Abstract

**Background/Aim:** Macrophage polarization and phenotypic switching of smooth muscle cells (SMCs) are multi-faceted events dominating atherosclerosis (AS) progression. TGF-β was proved to been one of the bridge on the crosstalk between macrophage and SMC. ShenLian (SL) was extracted from a potent anti-atherosclerotic formula. However, its exact mechanism rebalancing inflammatory microenvironment of AS remain largely unknown. Within the entirety of macrophage and SMC, this study investigated the pharmacological effects of SL on stabilizing atherosclerotic plaques.

**Methods:** The main components of SL were examined by high performance liquid chromatography. Co-culture and conditioned medium models of macrophage/SMC interactions were designed to identify the relationship between macrophage polarization and switching of SMC phenotypes. Flow cytometry, immunofluorescent staining, RT-PCR, western blotting, and ELISA were used to determine the expression of molecules relating to AS progression. An atherosclerosis animal model, established by placing a perivascular collar on the right common carotid artery in ApoE^−/−^ mice, was used to investigate whether TGF-β is the key molecular mediator of SL in crosstalk between macrophage and SMC. Plaque size was defined by nuclear magnetic resonance imaging. Key markers related to phenotypic transformation of macrophage and SMC were determined by immunohistochemical staining.

**Results:** Results revealed that, accompanied by rebalanced M2 macrophage polarization, SL supported SMC phenotypic transformation and functionally reconstruct the ECM of plaques specifically in macrophage-SMC co-cultural model. Molecularly, such activity of SL closely related to the activation of STAT3/SOCS3 pathway. Furthermore, in co-culture system, up-regulation of α-SMA induced by SL could neutralized by 1D11, a TGF-β neutralizing antibody, indicating that SL mediated Macrophage-SMC communication by enhancing TGF-β. In the AS model constructed by ApoE^−/−^ mice, effects of SL on phenotypic transformation of macrophage and SMC has been well verified. Specific blocking of TGF-β largely attenuated the aforementioned effects of SL.

**Conclusion:** Our findings highlighted that TGF-β might be the responsive factor of SL within macrophage and SMC communication. This study revealed that crosstalk between macrophage and SMC forms a holistic entirety promoting atherosclerotic plaque stability.

**GRAPHICAL ABSTRACT:**
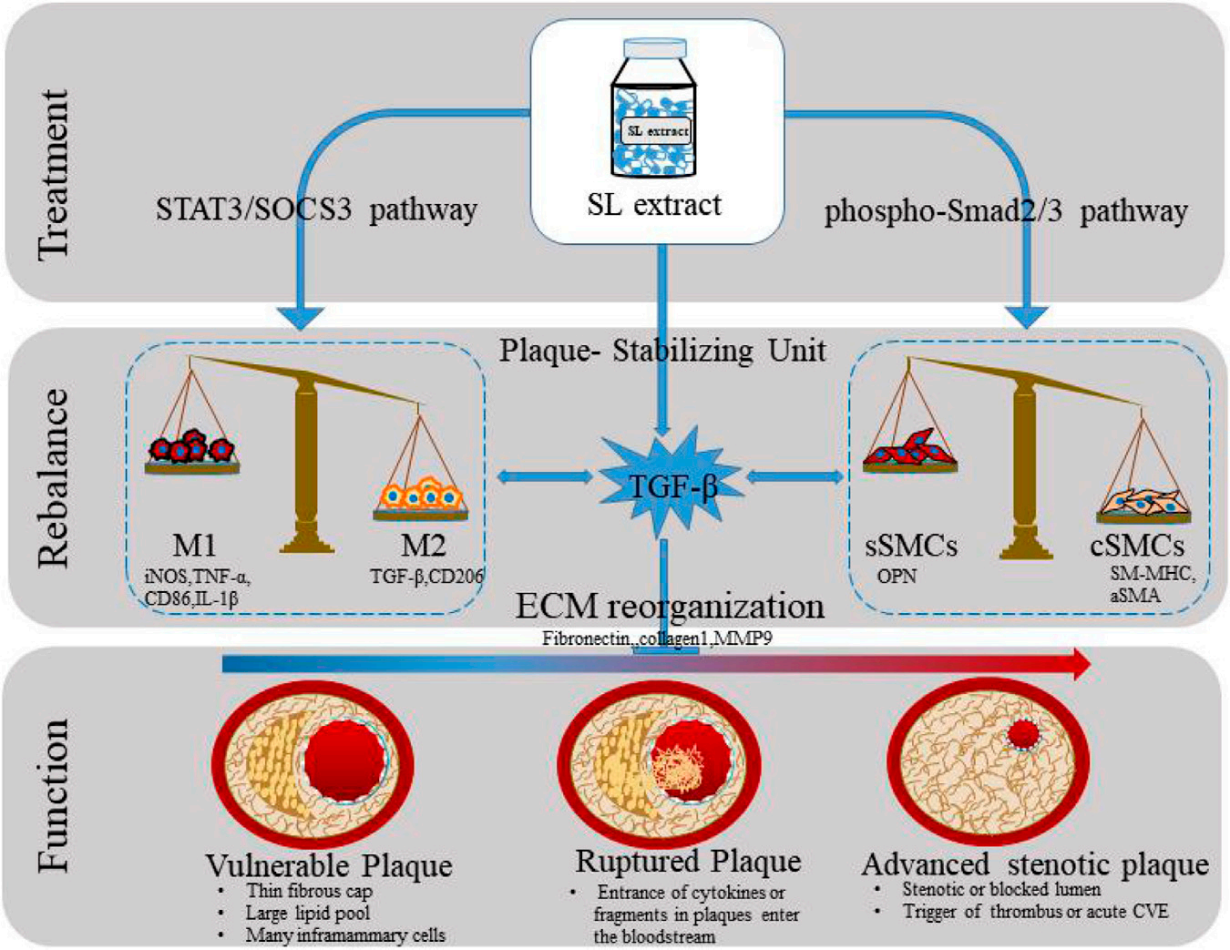
The graphic summary for stabilizing atherosclerotic plaques study of SL.

## Introduction

Cardiovascular diseases (CVDs) caused by atherosclerosis (AS) are the top-ranking global disease in terms of annual mortality (Moss et al., 2016). During the advanced stages of CVDs, the rupture of AS plaques can act as a major trigger for acute cardiovascular events (Badimon and Vilahur, 2014; Gistera and Hansson, 2017). Moreover, autopsy studies have shown that ruptured plaques are extremely thin and are infiltrated by large numbers of inflammatory cells (Badimon and Vilahur, 2014). Macrophage and smooth muscle cells (SMCs) are mainly responsible for regulation of inflammation and the stability of the fiber caps on plaques. (Gomez and Owens, 2012; Tabas and Bornfeldt, 2016). These two major cellular components of the fibrous cap on atherosclerotic plaques possess remarkable effects on phenotypic plasticity and play a pivotal role in preventing their rupture (Tabas and Bornfeldt, 2016; Harman and Jørgensen, 2019). Consequently, there is an urgent need to identify the precise function and interactions of macrophage and SMC with respect to atherosclerotic plaques.

As the main cellular component of the plaque fibrous cap, SMC often show a physiological trend to adopt a contractile phenotype, thus leading to normal vasoconstrictive activity (Jaminon et al., 2019). However, when blood vessels are damaged or stimulated, SMC is converted into a synthetic phenotype that is hypertrophic and promotes proliferation and migration, and reduces vascular compliance (Mathieu et al., 2020). Pathology-based studies have also documented that synthetic SMC dominates in the fibrous caps of atherosclerotic plaques and create fibrous caps that are thinner and more unstable; collectively, these features can result in an acute coronary event (Tilstam et al., 2020). Maintaining the contractile phenotype of SMC and stabilizing the extracellular matrix (ECM) are important factors when considering how to stabilize atherosclerotic plaques.

Macrophage is involved in all stages of AS and perform a dual role in that they can regulate both anti- and pro-inflammatory signals (Tabas and Bornfeldt, 2016; Shapouri-Moghaddam et al., 2018; Yang et al., 2020). M1 macrophage, in their classic activation state, are characterized by elevated levels of pro-inflammatory activity and can functionally contribute to the phenotypic switch of SMC towards a synthetic state. This process can increase the likelihood of plaque rupture (De et al., 2014; Tucureanu et al., 2016). In contrast, M2 macrophage, an alternatively activated status, exhibit strong inflammatory activity and can promote SMC to adopt a contractile state to promote plaque stabilization (Koga and Aikawa, 2012). Transforming growth factor beta (TGF-β) is viewed a maker of M2 polarized macrophage (Toma and McCaffrey, 2012). In addition, TGF-β is also a potent regulator of the synthesis and degradation of the ECM in an SMC-dependent manner (Goumans et al., 2018). Therefore, we propose that, by targeting TGF-β, bio-activity of macrophages could support the SMC phenotypic transformation and functionally reconstruct the ECM which would be beneficial for AS plaque stabilization, which is a useful therapeutic target for intervention in patients with AS.

Traditional Chinese medicine (TCM) considers atherosclerosis as the“chest bi-impediment” or “cardiac pain” induced by “Blood Heat” and “Blood Stasis” (Li, 2017; Zhang et al., 2020). After AS plaque has formed, TCM treatment of AS should be based on principle of clearing away heat and removing blood stasis. ShenLian (SL), extracted from *Salvia miltiorrhiza Bunge* and *Andrographis paniculata* which are the fully proved anti-atherosclerotic TCM formula (Guo et al., 2016; Guo et al., 2020). *Salvia miltiorrhiza Bunge* endowed with potent ability to promote blood circulation and remove blood stasis (Chen and Chen, 2017). *Andrographis paniculata* has excellent effects including clearing away heat, detoxifification, and purging fire of blood (Wu et al., 2018; Jiang et al., 2021). These functions of SL beneficial for prevent and treat atherosclerosis and cardiovascular diseases (Zhou et al., 2013; Guo et al., 2016). In our previous studies, we used high performance liquid chromatography (HPLC) to confirm the identity of the three main components (Andrographolide, Tanshinone IIA, Salvianolic acid B) of SL and revealed that these three active components existed in a certain ratio (Guo et al., 2020). Notably, Danshen dripping pills, whose main active components were extracted from *Salvia miltiorrhiza Bunge*, have been widely applied in the clinical treatment of CVDs since introduction into the Chinese drug market in 1994, and its pills were the first traditional Chinese medicine to complete Phase III clinical trials by the United States Food and Drug Administration (Writing Group of Recommendations of Expert Panel from Chinese Geriatrics Society on the Clinical Use of Compound Danshen Dripping Pills, 2017; Jia et al., 2018). Modern pharmacological studies have indicated that SL has the effect on intervene to atherosclerosis involving reduction of the atherosclerotic plaque area, an increase of blood flow in the proximal carotid artery, and regulation of lipids levels in the blood (Li et al., 2011; Guo et al., 2016; Guo et al., 2020). This study aimed to elucidate whether SL could enhance the bio-activity of macrophages to support the SMC phenotypic transformation and functionally reconstruct the ECM which would be beneficial for AS plaque stabilization.

## Materials and Methods

### Preparation of SL Extract and Regents

*Salvia miltiorrhiza Bunge* and *Andrographis paniculata* were obtained from Beijing Tongrentang Co., Ltd. The taxonomic authenticity was identified by Professor Xirong He, who worked in the Institute of Chinese Materia Medica of the China Academy of Chinese Medical Sciences. The SL extract is composed of *Salvia miltiorrhiza Bunge* extract and *Andrographis paniculata* extract at a ratio of 15:9. The *Salvia miltiorrhiza Bunge* extract included two components, one extracted with ethanol (EtOH) under percolation and was then concentrated under reduced pressure, and the other was prepared by soaking in dilute EtOH, and purified using macroporous resins SP825. The *Andrographis paniculata* extract was prepared by soaking in dilute EtOH, and purified using macroporous resins SP825. The extraction rates of the water-soluble partial extract of *Salvia miltiorrhiza Bunge* was 2.27% and that of the fat-soluble extract was 1.31%. The extraction rate of *Andrographis paniculata* was 2.11%. The components from SL extract were Tanshinone IIA (3%), salvianolic acid B (38%), and andrographolide (20%) and were detected by HPLC (Guo et al., 2020 or Supplementary Material S1).

Rosuvastatin(P), as positive control drug, was purchased from AstraZeneca Pharmaceuticals (China) Co., Ltd. Phorbol-12-myristate-13-acetate (PMA) was obtained from Beijing Solarbio Technology Co., Ltd. Oxidized low density lipoprotein (ox-LDL) was purchased from Shenzhen Angyubio Co., Ltd. JSI-124 (Cucurbitacin I hydrate), the JAK2/STAT3 blocker, was purchased from Sigma Co., Ltd. (United States). 1D11, a murine antibody that neutralizes all three major active TGF-β isoforms, was also provided by Genzyme (Framingham, United States).

### AS Animal Models and Drug Administration

Male ApoE^−/−^ mice (*n* = 80, 8 weeks old, on C57BL/6 J mice genetic background), and male C57BL/6 J mice (*n* = 10, 8 weeks old) were purchased from HFK Bioscience Co. (Beijing, China). All mice were housed under specific pathogen-free conditions. The animal experiments were performed in accordance with the Guidelines for the Care and Use of Laboratory Animals and were approved by the local Laboratory Animal Ethics Committee of the Institute of Chinese Materia Medica of the China Academy of Chinese Medical Sciences (Beijing, China; reference number 201802014). All mice were allowed to acclimatize with a standard laboratory diet for 1 week.

The atherosclerotic model was established using perivascular constrictive silastic collars (0.3 mm in the inner diameter) that were positioned on the right carotid arteries of ApoE^−/−^ mice. Meanwhile, the right common carotid artery of the control C57BL/6 J mice were isolated without positioning the constrictive collar. All mice were anesthetized by peritoneal injection of pentobarbital sodium (at a dose of 50 mg kg^−1^) during surgery.

Oneweek after surgery, ApoE^−/−^ mice were randomly divided by weight into six groups (10 mice/group). Each group was treated orally: the model group (0.5% carboxymethylcellulose sodium); positive control (rosuvastatin, 1.3 mg kg^−1^ d^−1^); SL extract low, medium, and high groups (95, 190, and 380 mg kg^−1^ d^−1^, respectively); the TGF-β blocking group (pirfenidone, 250 mg kg^−1^ d^−1^); and the combination group (SL 190 mg kg^−1^ d^−1^ + pirfenidone, 250 mg kg^−1^ d^−1^). Based on the pharmacodynamics study results in our previous studies, we chose 95, 190, 380 mg kg^−1^ d^−1^ as the low, medium, and high doses (Guo et al., 2016). Rosuvastatin (1.3 mg kg^−1^·d^−1^, referred to as P) was chosen as a positive control drug because it is a commonly used anti-atherosclerotic drug (Kalanuria et al., 2012). The ApoE^−/−^ mice were fed with a high-fat diet containing 10% lard, 1% cholesterol, 10% egg yolk powder, and 79% basal diet. The C57BL/6 J mice in the control group were fed ordinary rodent chow for 10 weeks.

### Cell Culture

The human acute monocytic leukemia cell line THP-1 obtained from the American Type Culture Collection (United States) was cultured in RPMI 1640. The mice monocytic macrophage cell line RAW264.7 was purchased from China Infrastructure of Cell Line Resource (China) and human aortic smooth muscle cells (HVASMCs, BeNa Culture Collection, China) were cultured in DMEM, supplemented with 10% of FBS, 1% L-glutamine, streptomycin (100 μg ml^−1^), and penicillin (100 units ml^−1^) and incubated at 37°C in a 5% CO2 atmosphere.

Three days before the extraction of peritoneal macrophage, 8-week-old C57BL/6 J mice were injected with 1 ml of 3% thioglycolate medium (BD Biosciences, United States). Peritoneal macrophage was isolated by washing the peritoneal cavity with endotoxin-free phosphate buffered saline (PBS, Hyclone, United States) and cultured at 37°C in 5% CO2 in RPMI 1640 medium supplemented by 10% fetal bovine serum (FBS, Hyclone). Peritoneal macrophage cells (2 × 10^6^·per well) were seeded into six-well plates with 1 ml fresh medium and washed three times with PBS before treatment.

### Establishment of THP-1 and HVASMCs Co-culture System

We designed two co-culture systems using macrophage and SMC to detect fibrosis and inflammatory levels influenced by the SL extract.

#### Protocol for Co-culture Experiments

1) THP-1 culture: THP-1 cells were cultured in 24-well plates with the permeable support basket (0.4 µm pore size, Costar) and stimulated with PMA (50 μg ml^−1^, Solarbio, Chain) to induce their differentiation to mature macrophage. After full induction, the differentiated cells can adhere onto the upper chamber for the subsequent experiments.
2) HVASMC culture: HVASMC cells were seeded in 24-well plates and cultured for 24 h.
3) Establishment of the co-culture system: the co-culture system was established 24 h after seeding by transferring the upper basket was lined with differentiated Thp1 cells into the wells containing HVASMC.
4) Drug treatment: After the co-culture system were successfully established, the SL extract (5.0, 10.0, 20.0 μg ml^−1^) was added to the co-culture medium for 24 h in the absence or presence of ox-LDL (10 μg ml^−1^), and then the drug-containing medium was totally removed and replaced with the fresh medium (SL-free medium).

#### Protocol for the Conditional Medium Transfer Model

To exclude the direct influence of SL on SMC, the conditional medium transfer model was established as Figure 4A. THP-1 cell was seeded on six well plate and then stimulated with PMA (50 μg ml^−1^) and treated with SL (5.0, 10.0, 20.0 μg ml^−1^) for 24 h in the absence or presence of ox-LDL (10 μg ml^−1^). Next, the SL-containing medium was replaced with fresh medium (SL-free medium) for 24 h and then transferred to the HVASMC plates. The SL-free medium and HVASMC ware collected for detection of indicators.

### Flow Cytometry

The cells were collected by cell scraper and fixed with 100% methanol (5 min), and then incubated in 3% FBS in 0.1% PBS-Tween 20 for 1 h to permeabilize the cells and to block non-specific protein-protein interactions followed by the antibody (CD206: 1:100 dilution, Abcam, United States; CD86: 1:150 dilution, Santa Cruz, Dallas, TX, United States) for 1 h at room temperature. The secondary antibody used was Fluorescein (FITC)-conjugated IgG (H + L) (ZSGB-BIO, China) at 1:500 dilution for 30 min at room temperature. After staining, the cells were analyzed with a CD206+gate by flow cytometry (Beckman, United States).

### Western Blotting Analysis

As described previously (Li Q et al., 2016), total protein was extracted using RIPA lysis buffer (Beyotime, China) containing 20 mM Tris HCl, pH 7.4, 150 mM NaCl, 1 mM EDTA, 1 mM EGTA, 1 mM PMSF, and 1% Triton X-100. The concentration of the protein samples was detected by the BCA Protein Quantitative kit (Beyotime, China). The same amounts of denatured protein samples were separated by sodium dodecyl sulfate-polyacrylamide gel electrophoresis (SDS-PAGE) and the gels were electrically blotted onto a PVDF membrane (Millipore, United States). Membranes were blocked with 5% skim milk in Tris-buffered saline containing 1% Tween 20 (TBST) for 2 h at room temperature. Primary antibodies included iNOS (1:1000 dilution, Abcam, United States ), phosphorylated (phospho)-JAK2 (1:2000 dilution, Abcam), JAK2 (1:100 dilution, Bioss Antibodies, China), phospho-STAT3 (1:1000 dilution, Abcam), total-SOCS3 (1:1000 dilution, Abcam, United States), Fibronectin (1:2000 dilution, Proteintech, China), α-SMA (1:4000 dilution, Proteintech, China), phospho-Smad2/3 (1:800 dilution, Abcam), Smad2/3 (1:800 dilution, Bioss Antibodies, China). Then the membrane was washed for three times by TBS-T and incubated with secondary antibody conjugated with horseradish peroxidase (HRP). The aim bands were visualized using chemiluminescence detection reagents (Thermo, United States). β-actin (ACTB) or GDPDH was used as the internal control.

### Enzyme-Linked Immunosorbent Assay

The cytokines TGF-β, tumor necrosis factor (TNF)-α, and interleukin (IL)-1β concentration from culture supernatants samples were measured by the enzyme-linked immunosorbent assay (ELISA) (Dakewei, China) method. The TNF-α, IL-1β, and TGF-β ELISA kits performed in this study were “Sandwich” kits which possess potent capacity for quantitatively measuring TNF-α, IL-1β, and TGF-β present in serum and culture media. The manufacturer’s instructions were precisely followed. The concentration of the target molecules in the sample was calculated, after making suitable correction for dilution, according to their standard curve.

### RT-PCR Assay

As described previously (Chen et al., 2016), mRNA levels of Collagen1, OPN, SM-MHC, α-SMA were determined by the reverse transcriptase-polymerase chain reaction (RT-PCR) assay. Total RNA was isolated from cells using TRIzol reagent (Life Technologies, United States). The detailed steps of the RT-PCR assay were performed in accordance with the manufacturer’s instructions (Thermo Fisher Scientific, United States). GAPDH was loaded as the internal control. Forward and reverse primer sequences are shown in Table 1. The amount of RNA obtained using agarose gel electrophoresis and the results were visualized and photographed using a UV transilluminator.

**TABLE 1:** Oligonucleotide primers used for RT-PCR.

Gene	Primer (5'→3′)	Annealing temperature (°C)	Length (bp)
GAPDH	Sense: CTA​AGG​CCA​ACC​GTG​AAA​AG	53	492
	Anti-sense: ACC​AGA​GGC​ATA​CAG​GGA​CA		
OPN	Sense: TGA​TGG​CCG​AGG​TGA​TAG​TG	53	210
	Anti-sense: CCAATCAGAAGGCGCGTT		
α-SMA	Sense: TGA​GCG​TGG​CTA​TTC​CTT​CGT	52	105
	Anti-sense: GCA​GTG​GCC​ATC​TCA​TTT​TCA​A		
SM-MHC	Sense: AAG​CCA​AGA​GCT​TGG​AAG​C	53	829
	Anti-sense: TCC​TCC​TCA​GAA​CCA​TCT​GC		
Collagen 1	Sense: ATG​GAT​TCC​AGT​TCG​AGT​ATG​GC	53	245
	Anti-sense: CAT​CGA​CAG​TGA​CGC​TGT​GG		

### Nuclear Magnetic Resonance Detection of Plaque Stability in ApoE^−/−^ Mice

Nuclear magnetic resonance (NMR) plays a significant role in the study of experimental atherosclerosis which can effectively assess luminal narrowing (van Bochove et al., 2010). In our study, mice were sent to the National Center for Nanoscience and Technology (Beijing) for NMR detection of the left and right carotid arteries (LCA and RCA) in the tenth week after modeling. The mice were firstly anesthetized with isoflurane gas. After the mice were anesthetized, the mice were connected to a gas anesthesia snorkel and placed in a 25 mm coil. A 7.05Tmagnetic field small animal NMR imager parameters: 25 mm coil, T1WI (TR 1328 ms, TE 5.53 ms); acquisition repetition times 1 time, layer thickness 0.5 mm, layer number 23, field of view (FOV) 25 mm, self-contained. The cervical coronal section of the mouse was scanned by a spiral echo sequence (SE). The obtained images were analyzed using ImageJ 1.4u (ImageJ, NIH, United States). The raw NMR image were presented at a ratio of 1:50. Then, we calculated the LCA and RCA areas (areas of each obtained NMR images divided by 2500).

### Sirius Red Staining and Immunohistochemistry

The samples to be subjected to Sirius red staining and immunohistochemistry (IHC) included the RCA within 1 cm (centering on the perivascular constrictive silastic collar (length, 3.5 mm). RCAs were fixed in 4% paraformaldehyde (pH 7.0–7.4) and then sliced into 4 μm sections for pathological evaluation. Sirius red staining (Solarbio, China) was performed according to the manufacture’s instruction for collagen staining of vascular plaque fibrous cap. Images were collected by an optical microscope graphic analysis system (BX51, Olympus, Japan). Quantification of collagen was determined with ImageJ 1.4u (ImageJ, NIHby measuring the ratio of the optical density (OD) of Sirius red staining to that of the aorta.

RCAs were sliced into 4 μm paraffin sections for pathological evaluation. Samples for IHC were subjected to heat-induced epitope retrieval and incubated with corresponding antibodies including CD86 (1:100 dilution, Santa Cruz, Dallas, TX, United States), CD206 (1:150 dilution, Abcam), α-SMA (1:2000 dilution, Proteintech, China), Fibronectin (1:200 dilution, Proteintech), MMP9 (1:200 dilution, Proteintech), at 4°C overnight, the samples for CD86 and CD206 subsequently labeled with HRP-conjugated immunoglobulin G (IgG), and α-SMA, Fibronectin, MMP9 labeled with biotin conjugated-second antibody at room temperature for 45 min. Finally, they were visualized by DAB and observed by upright microscopy (Carl Zeiss, Germany).

### Statistical Analysis

All imaging results were analyzed using ImageJ 2.0 (National Institute of Health, United States). Data were presented as the mean ± standard deviations (SD) and were processed using the statistical analysis software SPSS 17.0 (SPSS Inc., United States). Statistical analyses were performed by one-way analysis of variance (ANOVA), and an LSD post hoc test was used to show differences for each pair of factor levels. *p* < 0.05 was considered statistically significant.

## Results

### SL Extract Regulated Transformation of Macrophage Phenotype in an Inflammatory Injury Model

**FIGURE 1:**
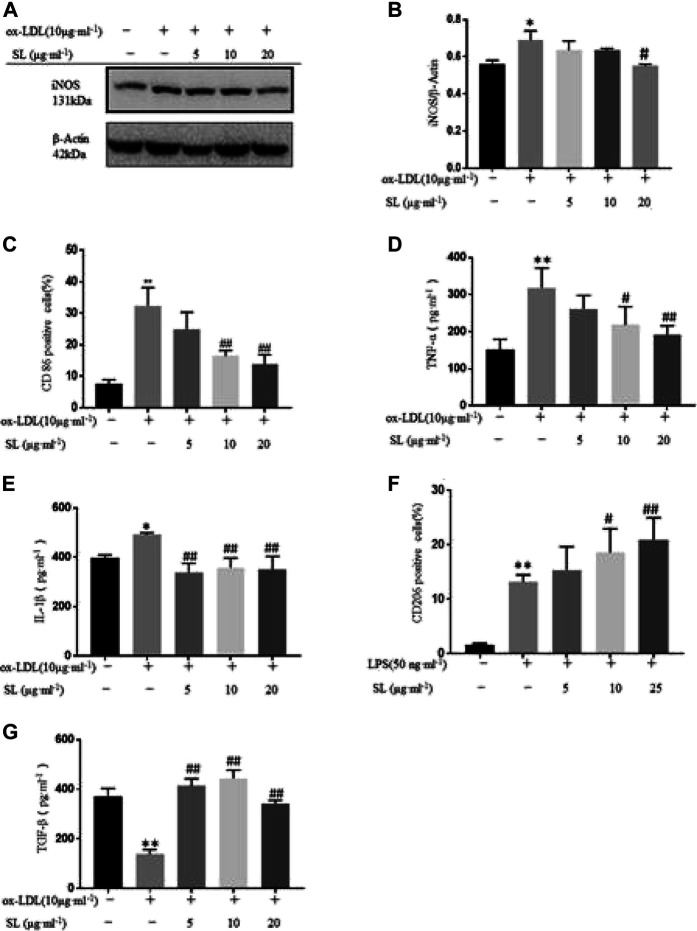
SL extract regulated transformation of macrophage phenotype in an inflflammatory injury model. **(A, B)** Western blotting verifification of M1 macrophage polarization related marker iNOS in cells treated with SL (from 5 to 20 μg ml-1) for 24 h in RAW264.7 cells in the absence or presence of ox-LDL, n=3. **(C)** Effect of SL on the expression of M1 marker CD86 in RAW264.7 by flflow cytometry. n=4 **(D, E)** Detection of M1 macrophage polarization related markers (TNF-α and IL-1β) by ELISA in supernatant of RAW264.7 cells treated with SL 24 h, n=3. **(F)** Effect of SL on expression of the M2 marker CD206 in RAW264.7 by flflow cytometry in the absence or presence of LPS (50 ng ml-1), n=6. **(G)** Detection of M2 macrophage polarization related marker (TGF-β) by ELISA in supernatant of RAW264.7 cells treated with SL 24 h, n=3. *p < 0.05 and **p < 0.01 compared to untreated group, and #*p* < 0.05 and ##*p* < 0.01 compared to control groups treated with model group.

Herein, we identified the effects of SL on transformation of macrophage phenotype in the inflammatory injury model. First, we examined the effects of SL on the expression of the M1 marker CD86 and M2 marker CD206 *in vitro* by flow cytometry. The results in Figures 1C,F showed that SL significantly inhibited expression of CD86 (Figure 1C) and facilitated the expression of CD206 (Figure 1F). Secondly, macrophage uptakes lipoproteins, particularly ox-LDL, and then become foam cells, which have been considered the main cells that contribute to the inflammation associated with atherosclerotic lesions. Using the ox-LDL as M1 priming factors, we further investigated effects of SL on macrophage polarization in the oxidized-lipid inflammatory injury model. Figures 1A,B show that ox-LDL increased iNOS protein expression and the SL extract reversed the trend. In the cell supernatant, the SL extract reduced the expression of TNF-α, IL-1β (Figures 1D,E), and increased that of TGF-β compared to cells induced by the ox-LDL group (Figure 1G). The results suggested that the SL extract could reverse the pathological polarization state of macrophage in the LPS- stimulated and ox-LDL- stimulated inflammatory injury model.

### Transformation of Macrophage Phenotype Induced by SL Involves STAT3/SOCS3 Pathway

**FIGURE 2:**
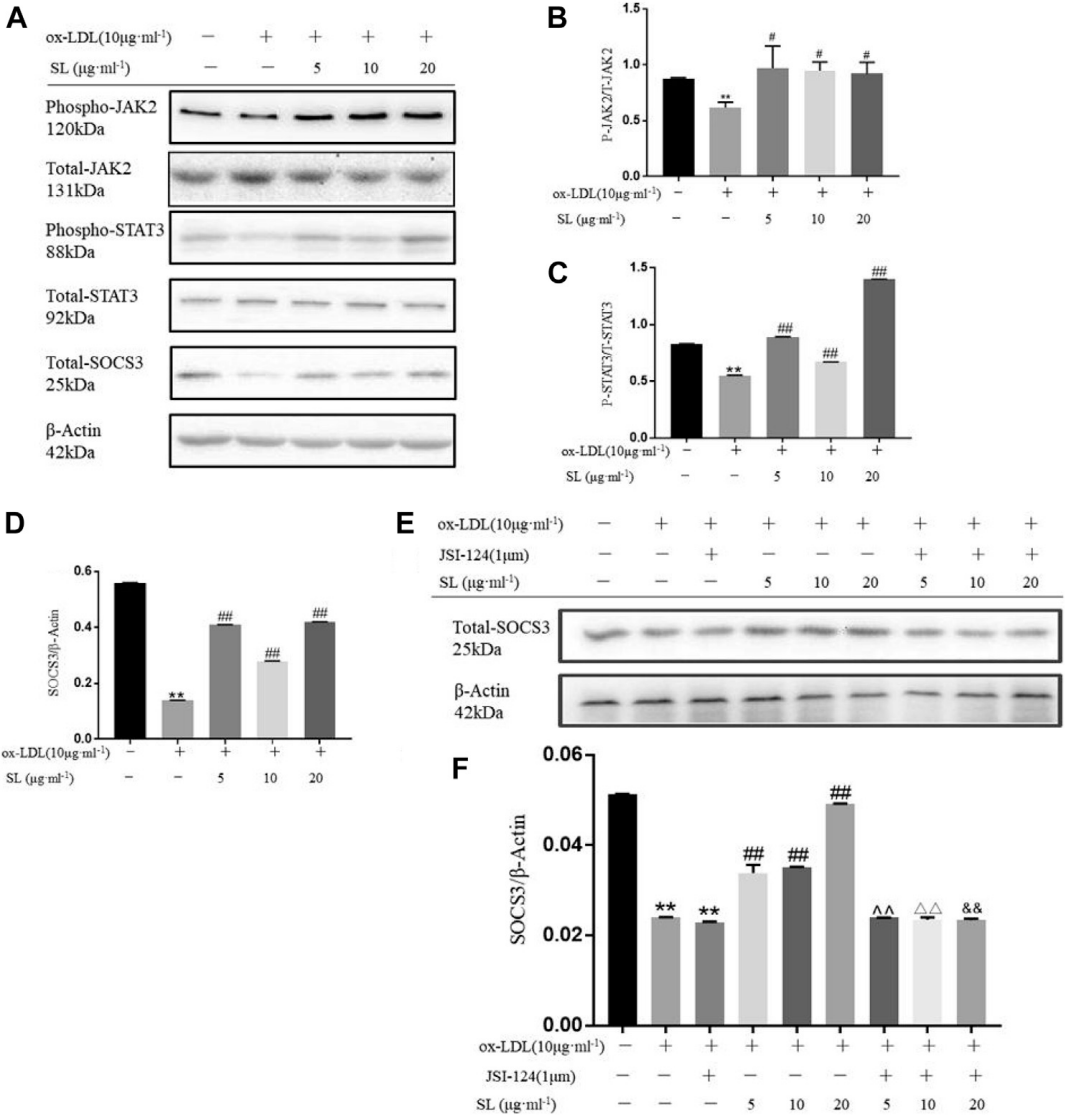
Transformation of macrophage phenotype induced by SL involves STAT3/SOCS3 pathway**. (A–D)** Detection of the phospho-JAK2, total-JAK2, phospho-STAT3, total-STAT3, total-SOCS3 and β-actin in SL-treated macrophages for 24 h, *n* = 3. **(E,F)** After specific blockading of JAK/STAT3 by JSI-124, total SOCS3 expression was detected by western blotting, *n* = 3. *p < 0.05 and **p < 0.01 compared to the untreated group, and #*p* < 0.05 and ##*p* < 0.01 compared to control groups treated with ox-LDL, ^ ^*p* < 0.01 compared with the SL-5 group, △△*p* < 0.01 compared with the SL-10 group, and && *p* < 0.01 compared with the SL-20 group.

Based on the functional influences of SL on macrophage polarization and TGF-β, we focused on the main regulatory pathway, the STAT3/SOCS3 pathway. As shown in Figures 2A–D, the SL extract can effectively promote the phosphorylation of JAK2, STAT3 and totle SOCS3 molecules compared with the ox-LDL group. Next, we blocked the JAK/STAT3 pathway by using its inhibitor JSI-124. The up-regulation of SOCS3 induced by SL was significantly neutralized (Figures 2E,F). Above the results suggested that the SL extract could effectively stimulate up-regulation in JAK2, STAT3, and SOCS3 phosphorylation. When the JAK2/STAT3 pathway was impeded, the efficacy of the SL extract was blocked, which indicated that the SL extract activated SOCS3 via the JAK2/STAT3 pathway.

### The SL Extract Increased the SMC Contractile Phenotype Both in Macrophage-SMC Co-cultures and When Exposed to Conditioned Media Models

**FIGURE 3:**
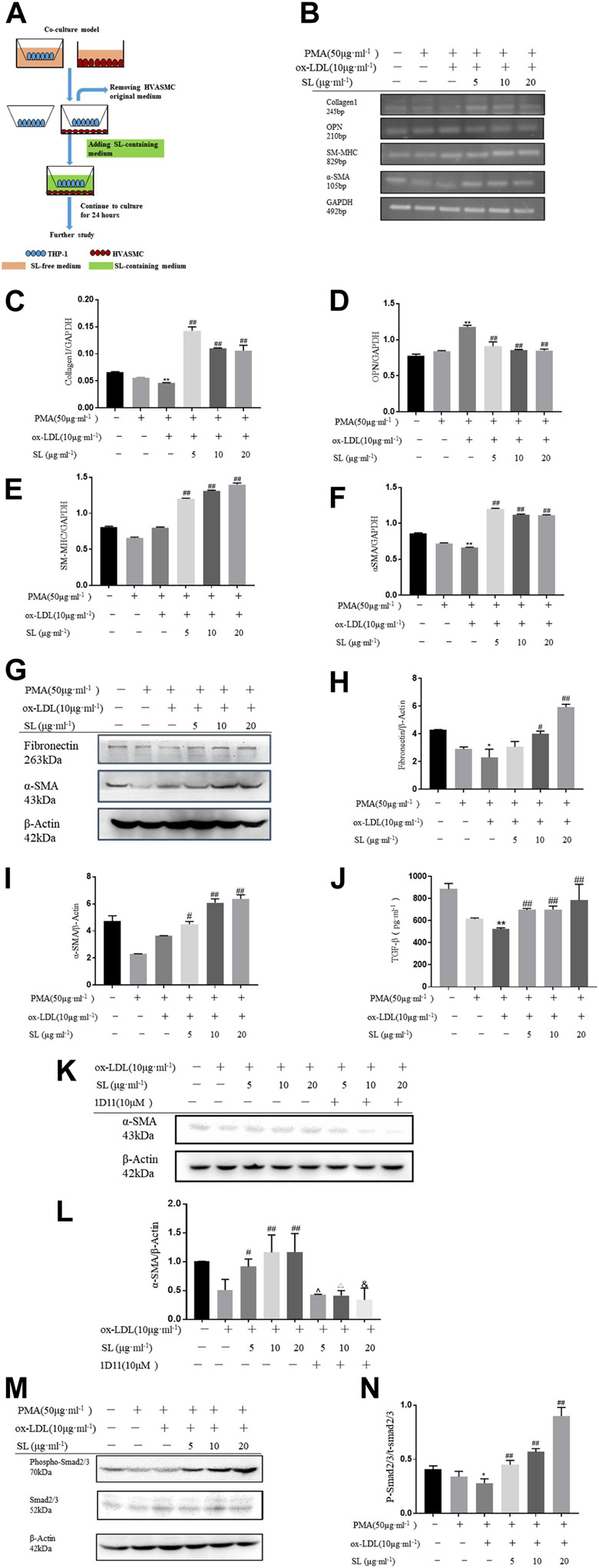
SL increases SMCs contractile phenotype in macrophage/SMC co-culture model. **(A)** *In vitro*, the protocol of co-culture model for efficacy identification of SL. **(B–F)** RT-PCR assay for ECM related marker (Collagen1), synthetic SMCs related markers (OPN), contractile SMCs related markers (SM-MHC, α-SMA) in HVASMCs from macrophage/SMCs co-culture model **(G–I)** Detection of the protein expression of Fibronectin, α-SMA and GAPDH treated with SL in HVASMCs from the macrophage/SMC co-culture model. **(J, K)** TGF-β was neutralized by TGF-β neutralizing antibody (1D11), Western blotting analysis for α-SMA protein expression **(L)** TGF-β levels detected by ELISA in the supernatant of macrophage/SMC co-culture model **(M, N)** *p*-SMA 2/3 normalized by Smad2/3 levels in the western blotting assay. *p < 0.05 and **p < 0.01 compared with the untreated group, and #*p* < 0.05 and ##*p* < 0.01 compared to control groups treated with ox-LDL, ^*p* < 0.05 compared with the SL-5 group, △*p* < 0.05 compared with the SL-10 group, and &*p* < 0.05 compared with the SL-20 group, *n* = 3.

**FIGURE 4:**
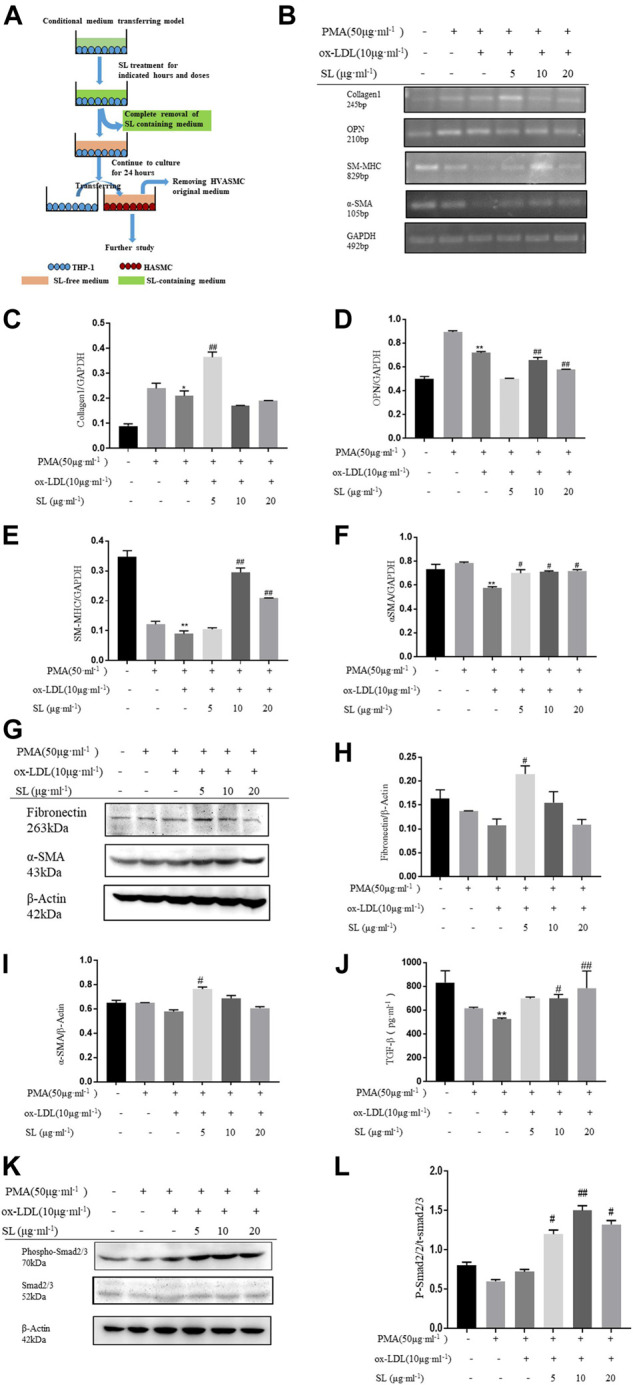
SL increases the SMC contractile phenotype in the macrophage/SMC conditional medium transferring model. **(A)** *In vitro*, the protocol of macrophage/SMC conditional medium transferring model for efficacy identification of SL. **(B-F)** RT-PCR assay for ECM related marker (Collagen1), synthetic SMC-related marker (OPN), contractile SMC-related markers (SM-MHC, α-SMA) in HVASMCs from the macrophage/SMC conditional medium transferring model. **(G-I)** Detection of the protein expression of fibronectin, α-SMA, and GAPDH treated with SL in HVASMCs from the macrophage/SMC conditional medium transferring model. **(J)** TGF-β levels detected by ELISA in the supernatant of the macrophage/SMC conditional medium transferring model. **(K, L)** p-Smad 2/3 normalized by Smad 2/3 level in western blotting assay. *p < 0.05 and **p < 0.01 compared with the untreated group, and #*p* < 0.05 and ##*p* < 0.01 compared to control groups treated with ox-LDL, n = 3.

To clarify whether SL regulates the interaction between macrophage and SMC, we established the macrophage-SMC co-culture and conditioned medium models, as shown in Figures 3A, 4A. The functional influences of SL treatment within the macrophage-SMC unit were molecularly analyzed. Specifically, the expression of ECM components was detected by evaluating collagen1 levels. In addition, the molecular markers representing SMC phenotypic changes were also quantified by the RT-PCR assay. As shown in Figures 3B,D, OPN, chosen as the marker for synthetic SMC, were significantly decreased in the SL treated groups. Conversely, we also observed enhanced mRNA expression of SM-MHC and α-SMA as well as that of α-SMA protein, which reflected the functional state of contractile SMC. In addition, we employed a TGF-β neutralizing antibody (1D11) (Tabe et al., 2013). in the co-culture system to investigate whether SL-induced SMC phenotypic switching was dependent on TGF-β released from M2 polarized macrophage. Consistent with our existing data, the result indicated that 1D11 neutralized the contractile SMC related marker (α-SMA) in the macrophage/SMC co-culture model. The above results may provide a molecular explanation of the induction of the contractile phenotypic of SL within the macrophage-SMC unit.

To specifically demonstrate that the phenotypic changes of SMC induced by SL were based on the integrative influences on the macrophage-SMC unit rather than on the direct effects on SMC alone, the conditioned medium transfer model was designed. In this model, SL extracts were first used to treat the macrophage alone. By replacing the SL-containing medium with drug-free medium at 24 h after treatment, we ensured that the direct effects of SL on the SMC could be clearly excluded. In accordance with our hypothesis, the results showed that the SL possesses the effect of up-regulating the mRNA of Collagen1, SM-MHC, α-SMA as well as the protein expression of fibronectin and α-SMA, and down-regulating mRNA of OPN.

Mechanistically, TGF-β/Smad2/3 is the primary pathway maintaining the SMC phenotypic balance and is responsible for the stability variation of AS plaques. In light of such recognition, our results indicated the activation states of this pathway in the presence of SL using the above-mentioned models. Figures 4L–N and Figures 1J–L show that the elevated concentrations of TGF-β can be effectively induced by SL exposure, and as a result, increased the phosphorylation level of Smad2/3 downstream of TGF-β.

### Specific Blocking of TGF-β Attenuated the Functional Plaque Stability Provided by SL in ApoE^−^/^−^ Mice

The silastic collars were positioned to the right carotid arteries of ApoE^−/−^ mice to establish the atherosclerotic plaque model. The effects of SL on acrophage-SMC unit and plaque stability was verified *in vitro*. Specific blocking of TGF-βof ApoE^−/−^ mice was used to investigate whether TGF-β is responsive molecular mediator of SL.

**FIGURE 5:**
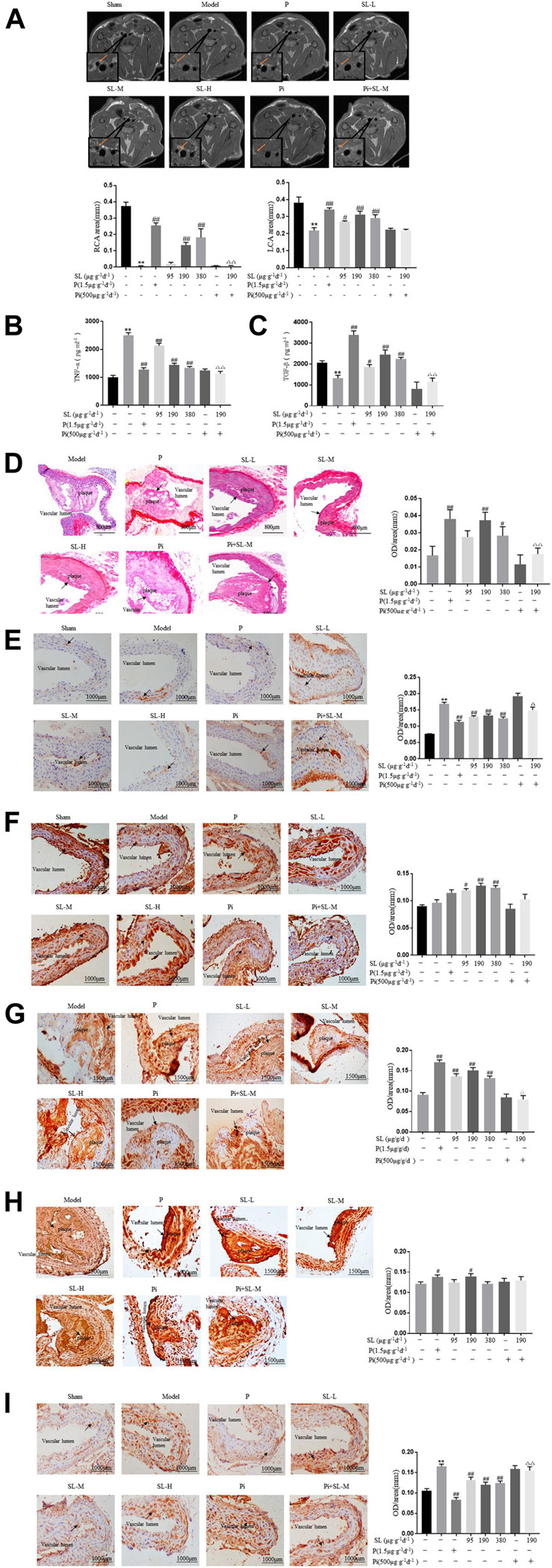
Specific blocking of TGF-β attenuated the functional plaque stability provided by SL in ApoE-/- mice. **(A)** Carotid artery coronal images captured by NMR and measurements of areas of the RCA and LCA. **(B, C)** TNF-α and TGF-β in the serum of treated were detected by ELISA. **(D)** Intravascular Sirius red, Scale bar, 800 μm. **(E, F)** IHC analysis and measurement of atherosclerotic aortic root, the expression of M1 macrophage polarization related marker CD86 and M2 macrophage polarization related marker CD206, Scale bar, 1000 μm. **(G, H)** IHC analysis and measurement of plaque, the expression level of contractile SMCs related markers α-SMA, ECM related marker Fibronectin, Scale bar, 1500 μm. **(I)** IHC analysis for MMP9, Scale bar, 1000 μm. *p < 0.05 and **p < 0.01 compared to the sham-treated group, and #*p* < 0.05 and ## *p* < 0.01 compared to model group, P = Rosuvastatin, Pi = Pirfenidone, n = 3.

Pharmacologically, as NMR image described (Figure 5A), in RCA of sham group, the inner wall of the blood vessel is clear, the shape is basically round and the lumen is transparent. In the model group, vascular lumen was almost blocked in RCA. While the LCA, the healthy carotid, the edge of the vessel wall has irregularities, and some highlights indicate the formation of small lipid plaques. By detecting the coronal view area, as shown in Figure 5A the areas of RCA and LCA in the model group were significantly reduced compared with the sham group. The vascular area in the Rosuvastatin group was significantly improved compared with the model group. Similarly, SL increased the coronal view area, especially in the middle and high dose groups.

In this model, to molecularly further identify the activity of SL on phenotypic transformation of macrophage and SMC. Representative factors of phenotypic transformation of macrophage and SMC were detected. As shown in Figure 5, the intervention of SL extract could effectively reduce the production of inflammatory cytokine TNF-α and increase the content of pro-inflammatory cytokine TGF-β in the mice serum (Figures 5A). Histopathologically, SL extract could increase the expression of M2 polarization maker CD206 (Figure 5F) and decrease the expression of M1 polarization maker CD86 (Figure 5E). Meanwhile, collagen is an indispensable and protective component for a stable plaque. Rosuvastatin and SL (SL-M and SL-H) was increased the collagen expression in plaque compared with model group (Figure 5D). We further verified the effects of SL on SMC phenotype switching and ECM. Contractile SMC related markers α-SMA, ECM related marker Fibronectin were increased by SL (Figures 5G,H). But as for the extracellular matrix, MMP-9 was reduced significantly in Rosuvastatin and SL extract groups (Figure 5I). This results ware in line with those obtained *in vitro* experiments.

Mechanistically, to further explore whether the TGF-β was the responsive molecular mediator of SL. Pirfenidone (Pi), the TGF-β blocker, was applied to the AS mice model. The Pi and SL-M combination group attenuated the effects of SL in regulating the RCA area of the arteries (Figure 5A), TNF-α (Figure 5B) and TGF-β (Figure 5C) levels, the intensity of collagen (Figure 5D), CD86 (Figure 5E), α-SMA (Figure 5G), and MMP-9 (Figure 5I) compared with the SL-M group.

Collectively, above data consistently demonstrated that SL treatment functionnally promotes macrophage-SMC unit and stabilizes atherosclerotic plaques which shown high correlation with TGF-β.

## Discussion

The stability of the atherosclerotic plaques is characterized by both dynamicity and plasticity, which contribute significantly to the progression of AS (Libby et al., 2011). A dense fibrosis plaque tends to be stable in the coronary artery. Conversely, when fibrous caps become thinner and contain a reduced number of collagen, there is a tendency for stable plaques to become much more vulnerable. Vulnerable plaques represent the trigger for the majority of acute cardiovascular events but are also the adjustable step in the progression of AS (Harman and Jørgensen, 2019). Therefore, maintaining the stability of plaques might extend the therapeutic time-window for patients who have already formed plaques. Furthermore, the development of drugs that can effectively target vulnerable plaques, especially during the late-phase of atherosclerosis, may make a significant contribution to the reduction of acute cardiovascular events. Previous studies have demonstrated that SL treatment possesses potent anti-atherosclerotic capabilities, at both theoretical and therapeutic levels (Li et al., 2011; Zhou et al., 2013; Guo et al., 2016; Li Y. J et al., 2016). *In vivo*, our results further showed that the collagen in plaque was up-regulated by SL (Figure 5D), meanwhile, the constituent of ECM was also changed in SL groups (Figures 5H,I). These results were consistently suggested that SL possessed potential effect on stabilizing atherosclerotic plaque.

Vulnerable plaque in AS was characterized by thin fibrous cap that is infiltrated by inflammatory cells and low VSMC density (Falk et al., 2013) (Koga and Aikawa, 2012) reported that macrophage may promote the activation and pro-atherogenic functions of SMC. In the AS model constructed by ApoE^−^/^−^ mice, we have evaluated the protein expression of phenotypic transformation of macrophage and SMC markers, which suggested that SL possesses a capacity for transferring phenotype of macrophage and SMC which would be beneficial for AS plaque stabilization (Figures 5E,F). Notably, besides the detection of markers in transformation of macrophage and SMC phenotype *in vivo*, we also have evaluated the protein expression of M1 and M2 makers in macrophage *in vitro* (Figure 2), which could further reflect the SL effects on M1/M2 polarization. Mechanically, the influence to macrophage of SL was closely correlated and likely attributable to the regulation of STAT3/SOCS3 pathway (Figure 3).

Although SL displayed the function on transformation SMC phenotype *in vivo* (Figure 5G), SL had no direct effect on protein expression of α-SMA (Supplementary Material S2), a contractile SMC related marker *in vitro*. Inspired by the interaction of macrophages and SMC and the effect of SL on macrophage, two co-culture systems were designed to identify whether SL regulate the crosstalk of the two cells to stabilize atherosclerotic plaque. The co-culture model represents an *in vivo* inflammatory microenvironment for SMC, while we excluded the direct influence of drugs on SMC by using the conditioned medium system. Confining within the macrophage-SMC communication, we observed the SL enhanced the bio-activity of macrophages to support the SMC phenotypic transformation and functionally reconstruct the ECM which would be beneficial for AS plaque stabilization. This pharmacological influence to macrophage of SL shares high similarity to and might be determined by M2 polarization in microenvironment from AS plaque.

SMC often reside close to macrophage in vascular lesions and are most likely to be influenced by cytokines released from polarized macrophages (Koga and Aikawa, 2012). Among all this factors, TGF-β has been proved to be the most dominant effect in pharmacological regulation (Vergallo and Crea, 2020). Our results showed that SL enhanced TGF-β expression in the supernatant of macrophage and two co-culture systems (Figures 1J, 2G, 4L), accompanied by increasing of contractilepic SMC(Figures 1, 4). TGF-β is often considered to have atheroprotective properties due to its role in tissue repair, particularly with regard to the biosynthesis of collagen and the reorganization of the ECM (Goumans et al., 2018). In our study, in the atherosclerotic model, a specific blocking or neutralizing of TGF-β largely attenuated SMC with a contractile phenotype and the anti-atherosclerotic function of SL. These results indicated that TGF-β might be the responsive factor of SL within macrophage and SMC communication.

The crosstalk between macrophage and SMC is a complex communication network involved multiple factors, dominated by but not limited to TGF-β, including platelet-derived growth factor, fibrogenic connective tissue growth factor, macrophage colony stimulating factor, MCP-1, IL-6 and so on (Hayashi et al., 1992; Vasudevan et al., 2003; Kang et al., 2012; Koga and Aikawa, 2012). As exemplified as growth factors (Hayashi et al., 1992; Kang et al., 2012), platelet-derived growth factor and fibrogenic connective tissue growth factor from macrophage were proved to foster the VSMC and promote its proliferation.

In conclusion, the present study demonstrated that SL stabilized atherosclerotic plaques, in which TGF-β might be the bridge factor within macrophage-SMC unit. In the future studies, we need a more systematic analysis to determine how SL enhances this unit to pharmacologically stabilize vulnerable plaques.

## Data Availability

All datasets generated for this study are included in the article/Supplementary Material.

## Abbreviations

AS, Atherosclerosis; CVD, Cardiovascular disease; ECM, Extracellular matrix; IL-1β, Interleukin-1β; JAK1, Janus kinases 1; MCP-1, Monocyte chemokine-1; MMPs, Matrix metalloproteinases; OPN, Osteopontin; ox-LDL, Oxidized low density lipoprotein; SMCs, Smooth muscle cells; α-SMA, α-smooth muscle actin; SOCS3, Suppressor of cytokine signaling 3; STAT3, Signal transducer and activator of transcription proteins; TGF-β, Transforming growth factor–β; TNF-α, Tumor necrosis factor-alpha.

## References

[B1] Badimon L. Vilahur G. (2014). Thrombosis Formation on Atherosclerotic Lesions and Plaque Rupture. J. Intern. Med. 276, 618–632. 10.1111/joim.12296 25156650

[B2] Chen W. Chen G. (2017). Danshen (Salvia Miltiorrhiza Bunge): A Prospective Healing Sage for Cardiovascular Diseases. Curr. Pharm. Des. 23, 5125–5135. 10.2174/1381612823666170822101112 28828985

[B3] Chen X. Li Q. Kan X. X. Wang Y. J. Li Y. J. Yang Q. (2016). Extract of Caulis Spatholobi, a Novel Blocker Targeting Tumor Cell Induced Platelet Aggregation, Inhibits Breast Cancer Metastasis. Oncol. Rep. 36, 3215–3224. 10.3892/or.2016.5184 27779702

[B4] De P. F. Staels B. Chinetti-Gbaguidi G. (2014). Macrophage Phenotypes and Their Modulation in Atherosclerosis. CIRC. J. 78, 1775–1781. 10.1253/circj.cj-14-0621 24998279

[B5] Falk E. Nakano M. Bentzon J. F. Finn A. V. Virmani R. (2013). Update on Acute Coronary Syndromes: the Pathologists' View. EUR. HEART. J. 34, 719–728. 10.1093/eurheartj/ehs411 23242196

[B6] Gistera A. Hansson G. K. (2017). The Immunology of Atherosclerosis. Nat. Rev. Nephrol. 13, 368–380. 10.1038/nrneph.2017.51 28392564

[B7] Gomez D. Owens G. K. (2012). Smooth Muscle Cell Phenotypic Switching in Atherosclerosis. Cardiovasc. Res. 95, 156–164. 10.1093/cvr/cvs115 22406749 PMC3388816

[B8] Goumans M. Ten D. P. (2018). TGF-β Signaling in Control of Cardiovascular Function. Csh. *Perspect. Biol.* 10, a22210. 10.1101/cshperspect.a022210 PMC5793760 28348036

[B9] Guo Y. Liu X. C. Wang Y. J. Li Q. Yang Q. Weng X. G. (2016). Effects of Shenlian Extract on Experimental Atherosclerosis in ApoE-Deficient Mice Based on Ultrasound Biomicroscopy. BMC. *Complement. Altern. Med.* 16, 469. 10.1186/s12906-016-1449-6 27846838 PMC5111256

[B10] Guo Y. Yang Q. Weng X. Wang Y. Hu X. Zheng X. (2020). Shenlian Extract against Myocardial Injury Induced by Ischemia through the Regulation of NF-Κb/iκb Signaling *Axis* . Front. Pharmacol. 11, 134. 10.3389/fphar.2020.00134 32210797 PMC7069067

[B11] Harman J. L. Jørgensen H. F. (2019). The Role of Smooth Muscle Cells in Plaque Stability: Therapeutic Targeting Potential. Brit. J. Pharmacol. 176, 3741–3753. 10.1111/bph.14779 31254285 PMC6780045

[B12] Hayashi K. Nishio E. Nakashima K. Amioka H. Kurokawa J. Kajiyama G. (1992). Role of Cholesterol-Accumulating Macrophages on Vascular Smooth Muscle Cell Proliferation. Clin. Biochem. 25 (5), 345–349. 10.1016/0009-9120(92)80014-8 1490297

[B13] Jaminon A. Reesink K. Kroon A. Schurgers L. (2019). The Role of Vascular Smooth Muscle Cells in Arterial Remodeling: Focus on Calcification-Related Processes. INT. J. MOL. SCI. 20, 5694. 10.3390/ijms20225694 PMC6888164 31739395

[B14] Jia C. Han S. Wei L. Dang X. Niu Q. Chen M. (2018). Protective Effect of Compound Danshen (Salvia Miltiorrhiza) Dripping Pills Alone and in Combination with Carbamazepine on Kainic Acid-Induced Temporal Lobe Epilepsy and Cognitive Impairment in Rats. Pharm. Biol. 56, 217–224. 10.1080/13880209.2018.1432665 29560767 PMC6130614

[B15] Jiang M. Sheng F. Zhang Z. Ma X. Gao T. Fu C. (2021). Andrographis Paniculata (Burm.f.) Nees and its Major Constituent Andrographolide as Potential Antiviral Agents. J. Ethnopharmacol. 272, 113954. 10.1016/j.jep.2021.113954 33610706

[B16] Kalanuria A. A. Nyquist P. Ling G. (2012). The Prevention and Regression of Atherosclerotic Plaques: Emerging Treatments. Vasc. Health Risk Manage. 8, 549–561. 10.2147/VHRM.S27764 PMC3459726 23049260

[B17] Kang S. W. Kim M. S. Kim H. S. Lee Y. J. Kang Y. H. (2012). Anti-atherogenic Activity of Wild Grape (Vitis Thunbergii) Extract Antagonizing Smooth Muscle Cell Proliferation and Migration Promoted by Neighboring Macrophages. Int. J. Mol. Med. 29 (6), 1137–1145. 10.3892/ijmm.2012.931 22407282

[B18] Koga J. Aikawa M. (2012). Crosstalk between Macrophages and Smooth Muscle Cells in Atherosclerotic Vascular Diseases. Vasc. Pharmacol. 57, 24–28. 10.1016/j.vph.2012.02.011 22402259

[B19] Li Y. J. Chen Y. You Y. Weng X. G. Yang Q. Ruan C. X. (2011). Effects of Shenlian Extracts on Atherosclerosis by Inhibition of the Inflammatory Response. J. Tradit Chin. Med. 31 (4), 344–348. 10.1016/s0254-6272(12)60016-8 22462243

[B20] Li Q. Wang Y. Xiao H. Li Y. Kan X. Wang X. (2016). Chamaejasmenin B, a Novel Candidate, Inhibits Breast Tumor Metastasis by Rebalancing TGF-Beta Paradox. Oncotarget 7, 48180–48192. 10.18632/oncotarget.10193 27374079 PMC5217010

[B21] Li Y. J. Ruan C. X. Yang Q. Weng X. G. Chen Y. Wang Y. J. (2016). Establishment of Vulnerable Plaque Model by Mast Cell Activation in Adventitia and Evaluation of Effectiveness of Intervention of Shenlian Tablet. Yao Xue Xue Bao 51, 1263–1270. 29898356

[B22] Li L. J. (2017). Professor Fang Zhuyuan's Experience in Treating Coronary Heart Disease with the Theory of "stasis and Heat Fighting. Inner Mongolia J. Traditional Chin. Med. 36, 53–54.

[B23] Libby P. Ridker P. M. Hansson G. K. (2011). Progress and Challenges in Translating the Biology of Atherosclerosis. Nature 473, 317–325. 10.1038/nature10146 21593864

[B24] Mathieu P. S. Fitzpatrick E. Di, Luca M. Cahill P. A. Lally C. (2020). Resident Multipotent Vascular Stem Cells Exhibit Amplitude Dependent Strain Avoidance Similar to that of Vascular Smooth Muscle Cells. Biochem. Biophys. Res. Commun. 521, 762–768. 10.1016/j.bbrc.2019.10.185 31706573

[B25] Moss J. W. E. Davies T. S. Garaiova I. Plummer S. F. Michael D. R. Ramji D. P. (2016). A Unique Combination of Nutritionally Active Ingredients Can Prevent Several Key Processes Associated with Atherosclerosis *In Vitro* . PLos. One. 11, e151057. 10.1371/journal.pone.0151057 PMC4780775 26950833

[B26] Shapouri-Moghaddam A. Mohammadian S. Vazini H. Taghadosi M. Esmaeili S. A. Mardani F. (2018). Macrophage Plasticity, Polarization, and Function in Health and Disease. J. Cel. Physiol. 233, 6425–6440. 10.1002/jcp.26429 PMC13482560 29319160

[B27] Tabas I. Bornfeldt K. E. (2016). Macrophage Phenotype and Function in Different Stages of Atherosclerosis. Circ. Res. 118, 653–667. 10.1161/CIRCRESAHA.115.306256 26892964 PMC4762068

[B28] Tabe Y. Shi Y. X. Zeng Z. Jin L. Shikami M. Hatanaka Y. (2013). TGF-β-Neutralizing Antibody 1D11 Enhances Cytarabine-Induced Apoptosis in AML Cells in the Bone Marrow Microenvironment. Plos. One. 8, e62785. 10.1371/journal.pone.0062785 23826077 PMC3695026

[B29] Tilstam P. V. Soppert J. Hemmers C. Harlacher E. Doring Y. van der Vorst E. P. C (2020). Non-activatable Mutant of Inhibitor of Kappa B Kinase Alpha (IKKalpha) Exerts Vascular Site-specific Effects on Atherosclerosis in Apoe-Deficient Mice. Atherosclerosis 292, 23–30. 10.1016/j.atherosclerosis.2019.10.023 31733453

[B30] Toma I. McCaffrey T. A. (2012). Transforming Growth Factor-β and Atherosclerosis: Interwoven Atherogenic and Atheroprotective Aspects. *CELL. TISSUE* . RES 347, 155–175. 10.1007/s00441-011-1189-3 PMC4915479 21626289

[B31] Tucureanu M. M. Butoi E. Gan A. Stan D. Constantinescu C. A. Calin M. (2016). Amendment of the Cytokine Profile in Macrophages Subsequent to Their Interaction with Smooth Muscle Cells: Differential Modulation by Fractalkine and Resistin. Cytokine 83, 250–261. 10.1016/j.cyto.2016.04.019 27180200

[B39] van Bochove G. S. Straathof R. Krams R. Nicolay K. Strijkers G. J. (2010). MRI-determined carotid artery flow velocities and wall shear stress in a mouse model of vulnerable and stable atherosclerotic plaque. MAGMA 23, 77–84. 10.1007/s10334-010-0200-4 20229088

[B32] Vasudevan S. S. Lopes N. H. Seshiah P. N. Wang T. Marsh C. B. Kereiakes D. J. (2003). Mac-1 and Fas Activities Are Concurrently Required for Execution of Smooth Muscle Cell Death by M-CSF-Stimulated Macrophages. Cardiovasc. Res. 59(3), 723–733. 10.1016/s0008-6363(03)00514-5 14499874

[B33] Vergallo R. Crea F. (2020). Atherosclerotic Plaque Healing. N. Engl. J. Med. 383, 846–857. 10.1056/NEJMra2000317 32846063

[B34] Writing Group of Recommendations of Expert Panel from Chinese Geriatrics Society on the Clinical Use of Compound Danshen Dripping Pills (2017). Recommendations on the Clinical Use of Compound Danshen Dripping Pills. Chin. Med. J. (Engl). 130, 972–978. 10.4103/0366-6999.204106 28397728 PMC5407045

[B35] Wu T. Peng Y. Yan S. Li N. Chen Y. Lan T. (2018). Andrographolide Ameliorates Atherosclerosis by Suppressing Pro-inflammation and ROS Generation-Mediated Foam Cell *Formation* . Inflammation 41, 1681–1689. 10.1007/s10753-018-0812-9 29948505

[B36] Yang S. Yuan H. Q. Hao Y. M. Ren Z. Qu S. L. Liu L. S. (2020). Macrophage Polarization in Atherosclerosis. Clin. Chim. Acta 501, 142–146. 10.1016/j.cca.2019.10.034 31730809

[B37] Zhang X. Q. Song Y. H. Jiang Y. Sun J. J. (2020). Treatment of Coronary Atherosclerotic Heart Disease from "stasis and Heat" Theory. Chin. J. Integr. Med. Cardio-Cerebrovascular Dis. 18, 2172–2174.

[B38] Zhou S. Y. Wang Y. H. Li Y. J. Yang Q. Gong Z. P. Ruan C. X. (2013). Effect of Shenlian Extracts on Blood Flow and Vessel Pathological Changes in Rabbits Carotid Atherosclerosis Model Induced by Low Shear Stress. Zhongguo Zhong Yao Za Zhi 38, 1595–1600. 23947145

