# Supplementary material for: ShenLian Extract Enhances TGF-β Functions in the Macrophage-SMC Unit and Stabilizes Atherosclerotic Plaques
Source: Front Pharmacol. 2021 May 28;12:669730. doi: 10.3389/fphar.2021.669730 (PMC8193129; doi:10.3389/fphar.2021.669730)
Supplement: Supplementary file 1 [file Presentation1.pdf]

1. HPLC chromatogram for determination of andrographolide content

1.1 Reagent

1.1.1 Reference Compound: Andrographolide (China Institute of Pharmaceutical and Biological Products, purity 98%, batch number: 110797-200307;).

1.1.2 Test Sample: *Andrographis paniculata* extract was identified from the whole herb of *Andrographis paniculata (Burm F.)* Nees.

1.2 Methods

Chromatographic conditions: Phenomenex Gemini wide PH C18 column (250×4.6 mm, 5 m); mobile phase: methanol-water (45/55, v/v), detection wavelength: 228 nm, column temperature: room temperature

1.3 Result

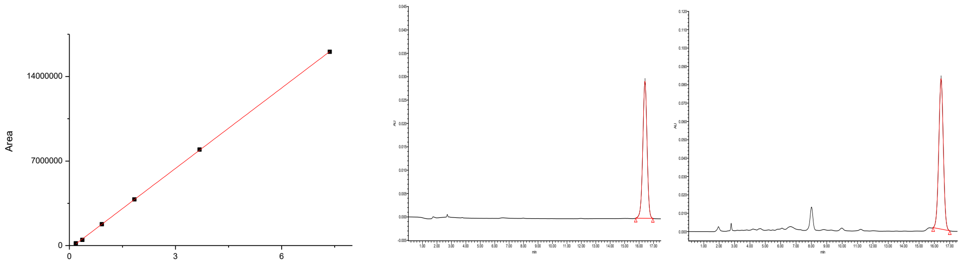

Andrographolide standard line   Reference Compound   Test Sample

Figure 1: HPLC chromatogram for determination of andrographolide content

2. HPLC chromatogram for determination of Tanshinone IIA content

2.1 Reagents

2.1.1 Reference Compound: Tanshinone IIA (China Pharmaceutical and Biological Products Inspection Institute, batch number: 110766-200417, purity above 98%);

2.1.2 Test Sample: the lip-soluble extract *Salvia miltiorrhiza* was identified from the dry rhizome of *Salvia miltiorrhiza Bge.*

2.2 Methods

Chromatographic conditions: Phenomenex Gemini C18 column (250×4.6 mm, 5 m), mobile phase: methanol/water (75/25, v/v), detection wavelength: 270 nm, column temperature: room temperature.

2.3 Results

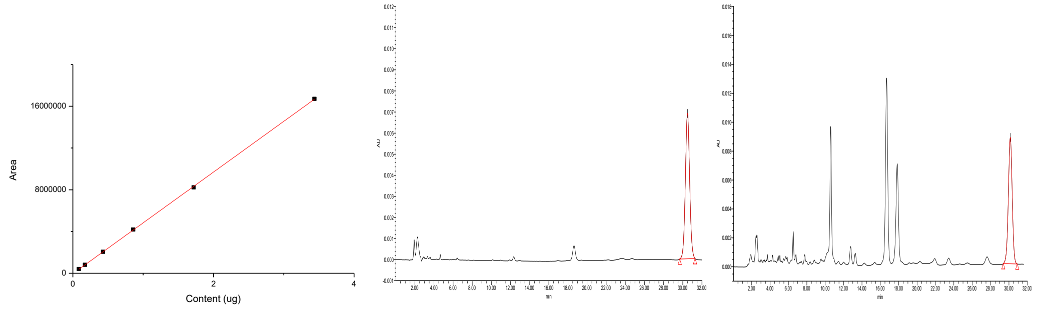

Tanshinone IIA standard line Reference Compound Test Sample

Figure 2: HPLC chromatogram for determination of Tanshinone IIA content

3. HPLC chromatogram for determination of Salvianolic acid B content

3.1 Reagents

3.1.1 Reference Compound: Salvianolic acid B (China Pharmaceutical and Biological Products Inspection Institute, batch number: 110766-200417, purity above 98%);

3.1.2 Test Sample: the water-soluble extract of *Salvia miltiorrhiza* was identified from the dry rhizome of *Salvia miltiorrhiza Bge.*,

2.2 Methods

Chromatographic conditions: Phenomenex Gemini C18 column (250×4.6 mm, 4 m), mobile phase: methanol/water (75/25, v/v), detection wavelength: 270 nm, column temperature: room temperature.

3.3Results

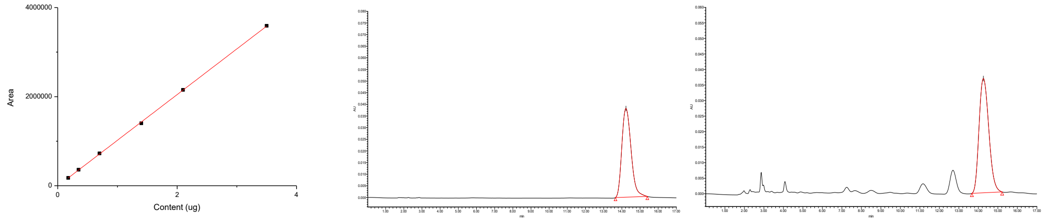

Salvianolic acid B standard line Reference Compound Test Sample

Figure 3: HPLC chromatogram for determination of Salvianolic acid B content
