# Supplementary material for: ShenLian Extract Enhances TGF-β Functions in the Macrophage-SMC Unit and Stabilizes Atherosclerotic Plaques
Source: Front Pharmacol. 2021 May 28;12:669730. doi: 10.3389/fphar.2021.669730 (PMC8193129; doi:10.3389/fphar.2021.669730)
Supplement: Supplementary file 2 [file Presentation2.pdf]

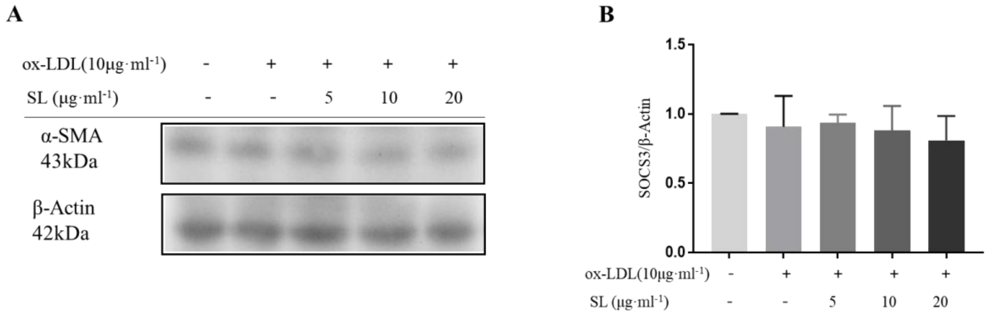

Figure 1: **Effects of SL on expression of α-SMA in HVASMC cells. n=3.** HVASMC cells were directly treated with SL (5.0, 10.0, 20.0 $\mu g \cdot mL^{-1}$) for 24 hours in the absence or presence of ox-LDL (10 $\mu g \cdot mL^{-1}$) and then the SMC phenotypic status was detected by protein expression of α-SMA, a contractile SMCs related marker.
